# Different response to hypoxia of adipose-derived multipotent cells from obese subjects with and without metabolic syndrome

**DOI:** 10.1371/journal.pone.0188324

**Published:** 2017-11-22

**Authors:** Wilfredo Oliva-Olivera, Isabel Moreno-Indias, Leticia Coín-Aragüez, Said Lhamyani, Juan Alcaide Torres, Sonia Fernández-Veledo, Joan Vendrell, Antonio Camargo, Rajaa El Bekay, Francisco José Tinahones

**Affiliations:** 1 Department of Clinical Endocrinology and Nutrition, Institute of Biomedical Research of Málaga (IBIMA), Hospital of Málaga (Virgen de la Victoria), University of Málaga (UMA), Málaga, Spain; 2 CIBER Fisiopatología Obesidad y Nutrición, Instituto de Salud Carlos III, Madrid, Spain; 3 Research Laboratory, Science School, University of Málaga (UMA), Málaga, Spain; 4 Hospital Universitari de Tarragona Joan XXIII, Institut d'Investigació Sanitària Pere Virgili, Universitat Rovirai Virgili, Tarragona, Spain; 5 CIBER de Diabetes y Enfermedades Metabólicas Asociadas (CIBERDEM), Instituto de Salud Carlos III, Madrid, Spain; 6 Lipids and Atherosclerosis Unit, IMIBIC/Reina Sofia University Hospital/University of Córdoba, Córdoba, Spain; University of Minnesota Medical Center, UNITED STATES

## Abstract

**Background/Objectives:**

Multiple studies suggest that hypoxia, together with inflammation, could be one of the phenomena involved in the onset and progression of obesity-related insulin resistance. In addition, dysfunction of adipose tissue in obese subjects with metabolic syndrome is associated with decreased angiogenesis. However, some subjects with a high body mass index do not develop metabolic abnormalities associated with obesity. The aim of the current study was to examine the neovascular properties of visceral adipose tissue-derived multipotent mesenchymal cells subjected to hypoxia (hypox-visASCs) from normal-weight subjects (Nw) and obese patients with metabolic syndrome (MS) and without metabolic syndrome (NonMS).

**Methods:**

This was a 2-year study to enroll subjects who underwent bariatric surgery or cholecystectomy. Eight patients who underwent either bariatric surgery or cholecystectomy (27 patients) participated in the study. Visceral adipose tissue samples from Nw, MS and NonMS subjects were processed by enzymatic digestion. VisASCs cultured under hypoxic conditions were characterized by tubule formation assay, ELISA, flow cytometry, migration rate, and qRT-PCR, and the effects of visASCs-conditioned medium on survival and endothelial cell tubule formation were evaluated.

**Results:**

Hypox-visASCs from NonMS subjects showed a greater capacity for tubule formation than hypox-visASCs from Nw and MS subjects. The lower percentage of CD140b^+^/CD44^+^ and CD140b^+^/CD184^+^ cells observed in hypox-visASCs from NonMS subjects compared to MS subjects was accompanied not only by a lower migration rate from the chemotactic effects of stromal cell derived factor 1α, but also by lower levels of NOX5 mRNA expression. While the levels of monocyte chemoattractant protein 1 mRNA expressed by hypox-visASCs correlated positively with the body mass index and waist circumference of the subjects, the concentration of vascular endothelial growth factor present in hypox-visASC-conditioned culture medium decreased significantly with increasing plasma glucose. The survival rate and tubules formed by endothelial cells cultured in hypox-visASC-conditioned medium decreased significantly with increasing homeostasis model assessment to quantify insulin resistance.

**Conclusions:**

Our results suggest that hypox-visASCs from NonMS subjects could promote healthy adipose tissue expansion, while hypox-visASCs from MS subjects appear to contribute to the decreased angiogenic potential and increased inflammation underlying adipose tissue dysfunction in obesity. Our results emphasize the importance of taking into account not only the BMI but also the metabolic profile of the subjects during the implementation of ASCs-based therapy to promote neovascularization.

## Introduction

*In vivo* experiments have confirmed that while subcutaneous adipose tissue expands by hyperplasia, visceral adipose tissue expands by hypertrophy [1]. It is thought that during expansion by hypertrophy, adipocytes can reach sizes that exceed the O_2(g)_ diffusion distance, thus generating hypoxic regions that activate angiogenic expansion [2–4]. It is likely that adipose tissue-derived multipotent mesenchymal cells (ASCs) actively participate in angiogenic expansion in regions of adipose tissue subjected to hypoxia, as it has been confirmed that a decrease in partial pressures of O_2(g)_ may increase their proliferation, migration and secretion of angiogenic cytokines [5–9] and contribute to the formation of new blood vessels *in vivo* [5; 10–13].

Hypoxia in adipose tissue is not always accompanied by a pro-angiogenic response [14] and may itself be an underlying cause of insulin resistance due to its ability to induce inflammatory responses in different cell types present in adipose tissue [15]. In fact, it has recently been observed that the deletion of hypoxia-inducible factor (HIF-1α) in adipocytes from high-fat diet-induced obese mice improves glucose tolerance and reduces macrophage infiltration [16]. On the other hand, although the expression of HIF1α in adipose precursor cells CD34^+^CD31^-^ was positively correlated with the body mass index (BMI) of the patients [17], the adipose tissue from obese subjects showed 44% lower capillary density, 58% lower vascular endothelial growth factor (VEGF) and greater macrophage infiltration than that of normal-weight (Nw) subjects [18].

While the insufficient availability of endothelial cells could be contributing to the failure of the angiogenic response in obesity [19], other experiments have shown the vascular density of omental adipose tissue to be positively correlated with BMI and waist circumference in subjects with severe obesity [20]. Thus, it seems likely that in the subjects whose adipose tissue retains its angiogenic ability, this adipose tissue could continue expanding without prejudice to the metabolic profile. We believe that variations in the neovascular response of ASCs to hypoxic conditions may be associated with differences in the angiogenic potential of adipose depots in obese subjects with metabolic syndrome (MS) and those without metabolic syndrome (NonMS). In the present study we set out to examine the neovascular properties, expression levels of proteins involved in cell redox balance and inflammatory cytokines in visceral adipose tissue-derived multipotent mesenchymal cells cultured under hypoxic conditions (hypox-visASCs) in subjects with different metabolic profiles.

## Materials and methods

### Subjects

The study included 35 participants recruited at the Virgen de la Victoria Clinical Hospital and the Civil Hospital (Malaga, Spain) who underwent bariatric surgery (eight morbidly obese patients) or cholecystectomy (27 patients) during the period 2012–2014. All subjects gave their informed consent to participate in the study, which was approved by the Ethics and Research Committee of Malaga (PI3-04/12). The study participants were less than 65 years of age, had no infectious disease, no acute cholecystitis, no type 2 diabetes nor drug treatment for this condition. Visceral adipose tissue biopsies (specifically, from the greater omentum) were obtained during the respective surgical procedures and the subjects with BMI ≥ 25 were grouped as MS or NonMS according to International Diabetes Federation criteria. Due to the limited availability of tissue, we were unable to conduct the experiments on all biopsies provided by all the study subjects. The number of experiments with cells from different donors (n) is specified in the respective assays. S1 Fig. depicts the entire study design.

### Isolation and expansion of stromal vascular fraction derived from visceral adipose tissue

Samples of visceral adipose tissue (greater omentum) were finely dissected and treated for 70 minutes at 37°C in a shaking water bath with type I collagenase enzyme solution (0.150% in PBS) supplemented with 1.0% BSA. The resulting tissue suspension was centrifuged for 10 minutes at 500×g and floating adipocytes were discarded by decanting. Subsequently, the stromal vascular fraction was filtered, centrifuged at 500×g for five minutes and the resulting cell pellet was resuspended in erythrocyte lysis buffer for ten minutes at room temperature. After washing, the pellet was resuspended and incubated for about 16 hours in growth medium consisting of DMEM/F12 supplemented with fetal bovine serum (FBS) (0.1 mL/mL), streptomycin (100 ug/mL), penicillin (100 U/mL) and L-glutamine (2 mM). The number of cells needed to perform the different assays was achieved by cell subculturing up to passage two or three and the proliferation associated with cell expansion took place at all times under standard culture conditions at 37°C in a humidified atmosphere and 5% CO_2(g)_, with two or three medium changes per week and up to 90% confluence.

### Tubule formation by hypox-visASCs

A total of 50 μL/cm^2^ growth factor-reduced matrigel (BD Biosciences, San Jose, CA, USA) was added to 48-well cell culture plates and incubated for 30 minutes at 37°C allowing the matrigel to solidify. VisASCs were resuspended in endothelial basal medium (EBM) (PromoCell, Sickingenstr, Heidelberg, Germany) supplemented with fetal calf serum (FCS) (0.05 mL/mL), seeded in duplicate at a density of 1 × 10^5^ cells/cm^2^ on gelled matrix, and incubated for six hours under conditions of 1% O_2(g)_, 94% N_2(g)_ and 5% CO_2(g)_ provided by hypoxia incubator (Thermo Scientific, Waltham, MA, USA). After six hours, phase-contrast fluorescence microscopy (Nikon, Japan) with a 4× objective was used to examine tubules, randomly selecting and photographing four fields per replicate. Subsequently, capillary-like structures were quantified using a Nikon NIS Elements image processor, considering as tubules those whose length exceeded four times the width.

### Collection of visASC-conditioned medium and ELISA

VisASCs were resuspended in growth medium, seeded in six-well plates at a density of 1.5 × 10^4^ cells/cm^2^ and maintained in standard culture conditions for ten days. Subsequently, this medium was replaced with EBM supplemented with FCS (0.05 mL/mL) and incubated under standard conditions or hypoxia (1% O_2(g)_). After 72 hours, conditioned medium from visASC culture was collected, centrifuged at 300×g for five minutes and reserved in aliquots at -80°C until use. ELISA kit (R&D Systems, Minneapolis, USA) was used to detect VEGF and hepatocyte growth factor (HGF) according to the manufacturer’s instructions. Data are expressed as mean ± standard error picograms of the cytokine per 10^6^ cells at the time of harvest.

### Immunophenotypic characterization by flow cytometry

VisASCs cultured for 72 hours under normoxic (normo-visASCs) or hypoxic conditions (hypox-visASCs) were characterized immunophenotypically by flow cytometry according to the cell surface markers CD44-fluorescein isothiocyanate (FITC) (Miltenyi Biotec, Bergisch Gladbach, Germany), CD140B-phycoerythrin (PE) (RD) and CD184-phycoerythrin cyanine 7 tandem fluorochrome (PE-Cy7) (BD). Briefly, visASCs were detached with trypsin/EDTA (pH 7.0–7.6), washed with PBS, resuspended in blocking buffer (PBS supplemented with 3.0% BSA), and incubated for ten minutes on ice. Aliquots of 1 × 10^5^ cells each were dispensed into polypropylene tubes, according to the manufacturer's instructions, and monoclonal mouse antibody solution against the respective cell surface markers, conjugated with their corresponding fluorochrome, was added. One tube was for labeling the corresponding IgG1-PE, IgG1-FITC (Miltenyi Biotec) and IgG1-PE-Cy7 (eBioscience, Santa Clara, California, USA) isotope controls. All tubes were incubated for 30 minutes on ice and protected from light. The cells were washed twice with blocking buffer then resuspended in 1000 μL of PBS to acquire 1 × 10^4^ events per tube using a CyAn^™^ ADP High-speed Analyzer (Beckman Coulter, Brea, California, USA)

### Chemotaxis assay of visASCs induced by stromal cell-derived factor 1α

VisASCs previously incubated for 72 hours under normoxic (21% O_2(g)_) or hypoxic (1% O_2(g)_) conditions were resuspended in EBM supplemented with FCS (0.05 mL/mL) and distributed in duplicate 3 × 10^4^ cells on the upper chamber of the 6.5 mm transwell plates with an 8 μm pore size (Corning, NY, USA). The lower chambers were occupied by either EBM supplemented with FCS (0.05 mL/mL) and 100 ng/mL of stromal cell-derived factor 1α (SDF1α) (R&D Systems, Minneapolis) or EBM supplemented with FCS (0.05 mL/mL) only. They were then allowed to migrate, incubating them for 24 hours under standard cell culture conditions. After this time, the non-migratory cells retained on the upper surface of the migration chamber were carefully removed using a cotton tip applicator and the cells that migrated to the undersurface of the transwell chamber were washed with PBS and fixed by incubating them with neutral buffered formalin for 15 minutes. Finally, the nucleus were labeled by incubating the cells with 4',6-diamidino-2-phenylindole (DAPI) (2.0 μg/mL) (Sigma-Aldrich, St. Louis, MO, USA) and phase-contrast fluorescence microscopy (Nikon, Japan) with a 10× objective was used to examine the migrated cells, randomly selecting and photographing five fields per replicate. The number of migrated cells per experimental condition was determined using a Nikon NIS Elements image processor and the migration rate under the chemotactic effects of SDF1α was calculated by dividing the number of cells detected in the presence of SDF1α by the number observed in the absence of SDF1α for cells previously cultured under hypoxia and normoxia.

### Gene expression by Real-time quantitative PCR

RNA from visceral adipose tissue (VAT) and cultured cells was isolated and purified using STAT-60 reagent (Amsbio, Abingdon, Oxon, UK) and reverse transcribed to cDNA using reverse-transcriptase enzyme (Transcriptor Reverse Transcriptase 20U/μL; 03531287001; Roche) in a 2720 Thermal Cycler (Applied Biosystems, Foster City, USA). Real-time quantitative PCR (RT-qPCR) was performed with 10 ng cDNA for NOX4 (Hs00418356_m1, NM_001143836.1), NOX5 (Hs00225846_m1, NM_001184779.1), Superoxide dismutase 2, mitochondrial (SOD2) (Hs00167309_m1, NM_000636.3), Interleukin 1β (IL1β) (Hs01555410_m1, NM_000576.2), Interleukin 8 (IL8) (Hs00174103_m1, NM_000584.3), Monocytes chemoattractant protein 1 (MCP1) (Hs00234140_m1, NM_002982.3), Transforming growth factor β1 (TGFβ1) (Hs00998133_m1, NM_000660.4), CD11C (Hs00174217_m1, NM_000887.4), CD163 (Hs00174705_m1, NM_004244.5). The amplifications were performed using a MicroAmp optical 96-well reaction plate (PE Applied Biosystems) on an Applied Biosystems 7500 Fast Real-Time PCR System (Applied Biosystems). RT-qPCRs were carried out for all genes using specific TaqMan gene expression assays and the procedure was performed as recommended by the manufacturer. Specific signals were normalized with respect to endogenous control ribosomal protein L13A (RPL13A) (Hs 04194366-g1, NM_001270491.1) to visASCs and 18S rRNA (4310893E, X03205.1) to VAT according to the 2^-ΔCt^ formula.

### Effect of visASC-conditioned medium on HUVEC survival and tubule formation

Human umbilical vein endothelial cells (HUVECs) (Promega, Madison, WI, USA) at passage 3 were seeded at a density of 1 x 10^4^ cells/cm^2^ in dark 96-well plates (Sigma) in EBM (Promocell) supplemented with FCS (0.05 mL/mL) and incubated for 24 hours under standard cell culture conditions. Subsequently, culture medium was replaced with 200 μL of hypox-visASC-conditioned medium and incubated for 72 hours under standard cell culture conditions. Finally, the dead cells were discarded by washing with PBS, and the surviving cells that remained adhered to the culture surface were reserved at -80 for at least one week for later quantification by the Cyquant Cell Proliferation kit (Invitrogen, Carlsbad, CA, USA). Cell viability was estimated by fluorescence intensity, by dividing the emitted fluorescence value in the wells where HUVECs were cultured in hypox-visASC-conditioned medium by the values emitted in the wells where HUVECs were cultured in unconditioned medium.

To determine the effects of the conditioned medium on HUVEC tubule formation, we coated 96-well plates (Corning) with 25 μL of growth factor-reduced matrigel and incubated them for 30 minutes at 37°C. 25 × 10^3^ HUVECs were dispersed into visASC-conditioned culture medium according to the respective experimental conditions of hypoxia or normoxia and seeded on gelled matrix. Cells were incubated under standard conditions for 24 hours, then capillary-like structures were examined as described above for visASCs. The rate of increase in tubule formation was calculated by dividing the mean length of the tubules produced by HUVECs cultured in hypox-visASC-conditioned medium by the mean length of those generated by the HUVECs cultured in normo-visASC-conditioned medium.

### Statistical analysis

The results were expressed as mean values ± SEM. The Shapiro-Wilk test was used to test for normality. Comparisons between more than two groups were performed using the nonparametric Kruskal-Wallis test and between two unpaired groups using the nonparametric Mann-Whitney U test. The correlation between variables was calculated with Spearman's rho. All the statistical analysis was done using SPSS (version 17.0; SPSS Inc, Chicago, IL). P values < 0.05 were considered statistically significant.

## Results

### Anthropometric characterization and metabolic profile of the subjects

Table 1 shows the clinical characteristics of the subjects. Both MS and NonMS subjects had a significantly higher BMI and waist circumference compared to Nw subjects. However, MS subjects showed greater deterioration in their metabolic profile because most of the metabolic variables evaluated in these patients differed significantly compared to at least one of the other two groups, with the exception of total cholesterol, systolic blood pressure, creatinine, and glutamic-oxaloacetic transaminase (GOT).

**Table 1:** Anthropometric and metabolic characteristics of the study participants.

	Nw (n = 6)	NonMS (n = 12)	MS (n = 17)
Cholecystectomy/Bariatric surgery	6/0	9/3	12/5
Age, years	41.50 ± 3.23	43.67 ± 2.12	43.47 ± 1.90
BMI, kg/m^2^	22.92 ± 0.55	34.54 ± 2.53***	36.27 ± 1.89***
Waist circumference, cm	79.50 ± 3.46	108.67 ± 4.96**	112.32 ± 3.91***
Waist-to-hip ratio	0.89 ± 0.01	0.90 ± 0.02	0.93 ± 0.02
HOMA-IR	2.01 ± 0.30	2.31 ± 0.26	4.48 ± 0.36***♯
Serum glucose, mmol/L	5.56 ± 0.16	5.13 ± 0.14*	6.01 ± 0.21††
Serum Insulin, pmol/L	55.35 ± 9.24	69.17 ± 7.64	118.27 ± 10.49**††
Triglycerides, mmol/L	1.40 ± 0.24	1.34 ± 0.12	2.47 ± 0.42†
HDL cholesterol, mmol/L	1.55 ± 0.12	1.28 ± 0.12	1.03 ± 0.08**
LDL cholesterol, mmol/L	2.66 ± 0.22	3.26 ± 0.51	3.60 ± 0.18*†
Total cholesterol, mmol/L	4.87 ± 0.21	4.96 ± 0.41	5.66 ± 0.30
Systolic blood pressure, mm Hg	122.50 ± 4.09	125.42 ± 3.25	127.65 ± 3.77
Diastolic blood pressure, mm Hg	79.33 ± 2.76	74.58 ± 2.48	82.35 ± 2.18†
Serum creatinine, μmol/L	67.18 ± 5.30	68.95 ± 6.19	79.56 ± 5.30
Serum urea, mmol/L	9.25 ± 0.71	9.91 ± 0.80	11.76 ± 0.76*
Uric acid, μmol/L	206.41 ± 14.87	254.70 ± 16.66*	303.97 ± 18.44**
Apolipoprotein A, g/L	1.82 ± 0.13	1.56 ± 0.07	1.46 ± 0.08*
Apolipoprotein B, g/L	0.87 ± 0.04	0.96 ± 0.10	1.16 ± 0.07*
GGT, μkat/L	0.35 ± 0.04	0.40 ± 0.05	1.07 ± 0.35**†
GOT, μkat /L	0.25 ± 0.04	0.41 ± 0.08	0.32 ± 0.03
GPT, μkat /L	0.35 ± 0.04	0.53 ± 0.09	0.63 ± 0.06**

Values are means ± SEM. Significant differences with respect to Nw individuals, Mann-Whitney:

* P < 0.05;

** P < 0.01;

*** P < 0.001.

Significant differences between MS and NonMS patients, Mann-Whitney:

^**†**^ P < 0.05;

^**††**^ P < 0.01;

^**♯**^ P < 0.001.

Nw: normal-weight subjects; NonMS: obese subjects without metabolic syndrome; MS: obese subjects with metabolic syndrome; BMI: body mass index; HOMA-IR: homeostasis model assessment to quantify insulin resistance; HDL cholesterol: high-density lipoprotein cholesterol; LDL cholesterol: low-density lipoprotein cholesterol; GGT: γ-glutamyltransferase; GOT: glutamic oxaloacetic transaminase; GPT: glutamate pyruvate transaminase.

### Increased capacity for tubule formation under hypoxic conditions in visASCs from NonMS subjects

Because the expansion of adipose tissue is associated with generating hypoxic regions that activate angiogenic expansion [2–4], we examined the capacity of visASCs from subjects with different metabolic profiles to generate capillary-like structures under hypoxic conditions.

**Fig 1:**
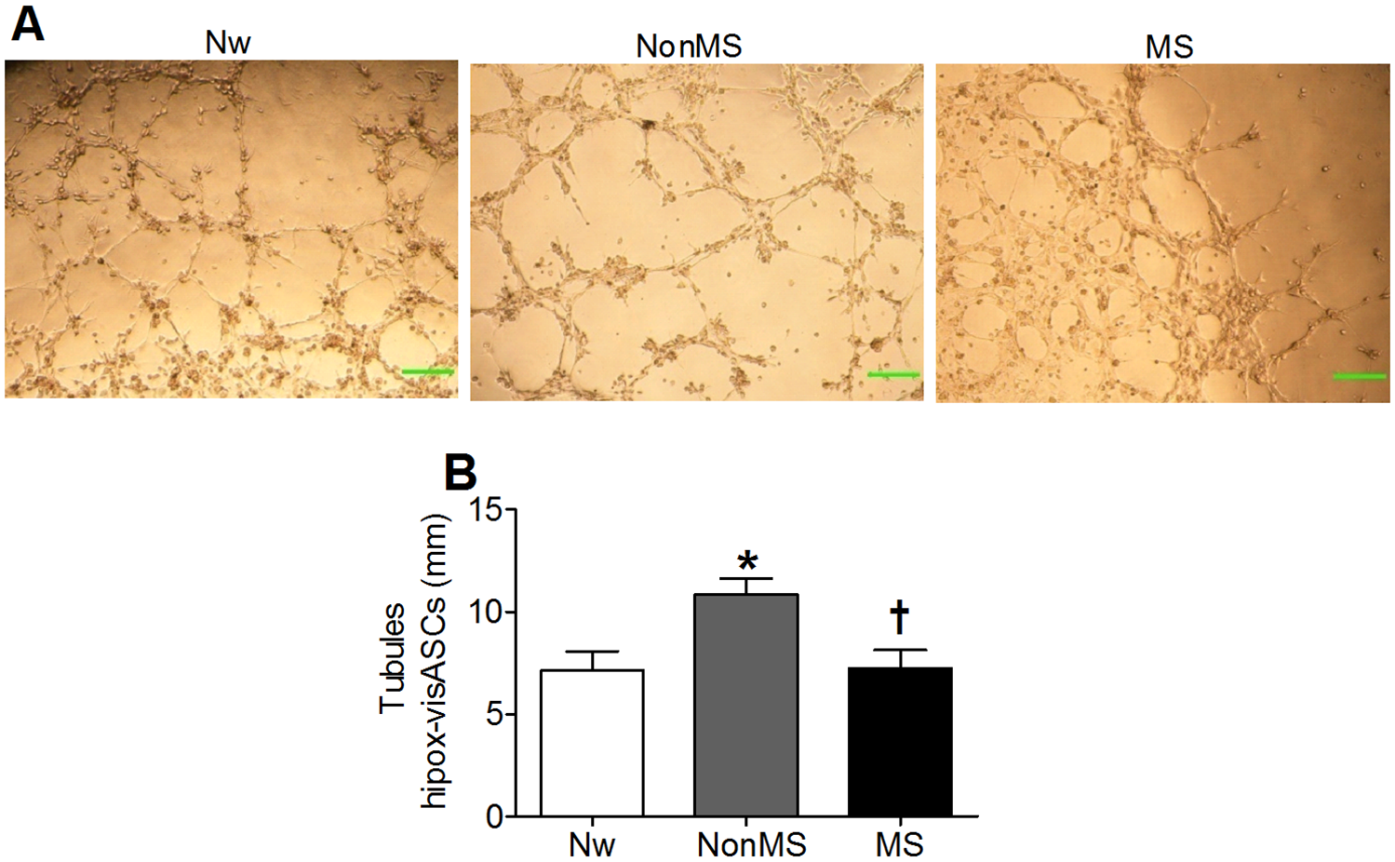
Capacity for tubule formation by hypox-visASCs. **A**: Generation of capillary-like structures by visASCs grown under hypoxic conditions (1% O_2(g)_) for six hours on 48-well plates coated with 50 μL/cm^2^ of growth factor-reduced matrigel. Bar = 250 μm. **B**: Values for the length of the tubules generated by hypox-visASCs from subjects grouped by metabolic profile. Bar = 250 μm. (Nw, n = 6; NonMS, n = 9; MS, n = 9). *, Significantly different results (Mann-Whitney test; P < 0.05) to those of Nw subjects; **†**, Significantly different results (Mann-Whitney test, P < 0.05) to those of NonMS subjects. Nw: normal-weight subjects; NonMS: obese subjects without metabolic syndrome; MS: obese subjects with metabolic syndrome; Hypox-visASCs: visceral adipose tissue-derived multipotent mesenchymal cells cultured under hypoxic conditions.

Tubule formation assay in growth factor-reduced matrigel confirmed the capacity of visASCs to generate capillary-like structures under 1% O_2(g)_ (Fig 1A) and revealed a significant increase in the length of capillary-like structures generated by visASCs from NonMS subjects compared to those formed by visASCs from the other two groups of subjects (Fig 1B).

### VisASCs from MS subjects showed an increased percentage of CD140b^+^ cells and an increased migration capacity under hypoxic conditions

Because CD140b and CD44 are cell-surface markers through which the perivascular localization of mesenchymal cells in multiple human organs has been defined [21–22] and considering the involvement of the CD184 receptor in the chemotactic effects of SDF1α on human ASCs subjected to hypoxia [6], we subsequently quantified the percentage of ASCs expressing these receptors.

**Fig 2:**
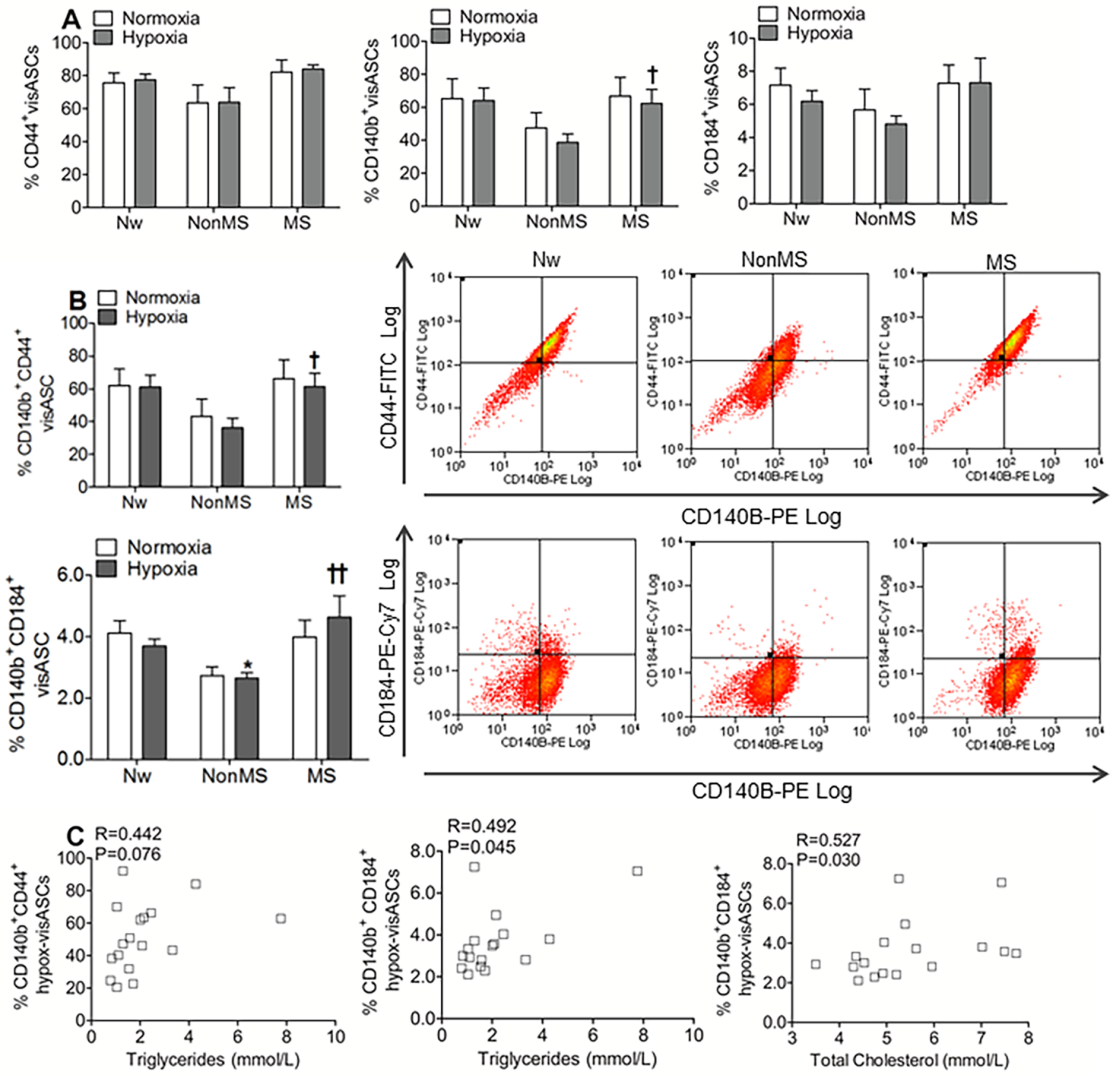
Immunophenotypic characterization of visASCs cultured for 72 hours under 21% O_2(g)_ or 1% O_2(g)_. **A-B**: Percentage of visASCs that expressed CD44, CD140b and CD184 **(A)** and coexpressed CD140b CD44 or CD140b CD184 **(B)** according to the metabolic profile of the subjects. **C**: Correlation of percentage of CD140b^+^CD44^+^ and CD140b^+^CD184^+^ hypox-visASCs with plasma levels of triglycerides and total cholesterol in the patients. (Nw, n = 3; NonMS, n = 7; MS, n = 7). Significant differences with respect to Nw individuals, Mann-Whitney: * P < 0.05; Significant differences between MS and NonMS patients, Mann-Whitney: **†** P < 0.05; **††** P < 0.01. Nw: normal-weight subjects; NonMS: obese subjects without metabolic syndrome; MS: obese subjects with metabolic syndrome; Hypox-visASCs: visceral adipose tissue-derived multipotent mesenchymal cells cultured under hypoxic conditions.

Immunophenotypic characterization of visASCs cultured for 72 hours under normoxic and hypoxic conditions revealed a statistically significant decrease in the percentage of CD140b^+^ hypox-visASCs from NonMS subjects with respect to MS subjects (Fig 2A).

Compared with hypox-visASCs from MS and Nw subjects, hypox-visASCs from NonMS subjects also showed a lower percentage of cells coexpressing CD140b/CD44 and CD140b/CD184 (Fig 2B). Moreover, we note that the percentage of hypox-visASCs coexpressing CD140b/CD44 and CD140b/CD184 was associated with a worsening lipid profile in the patients (Fig 2C).

**Fig 3:**
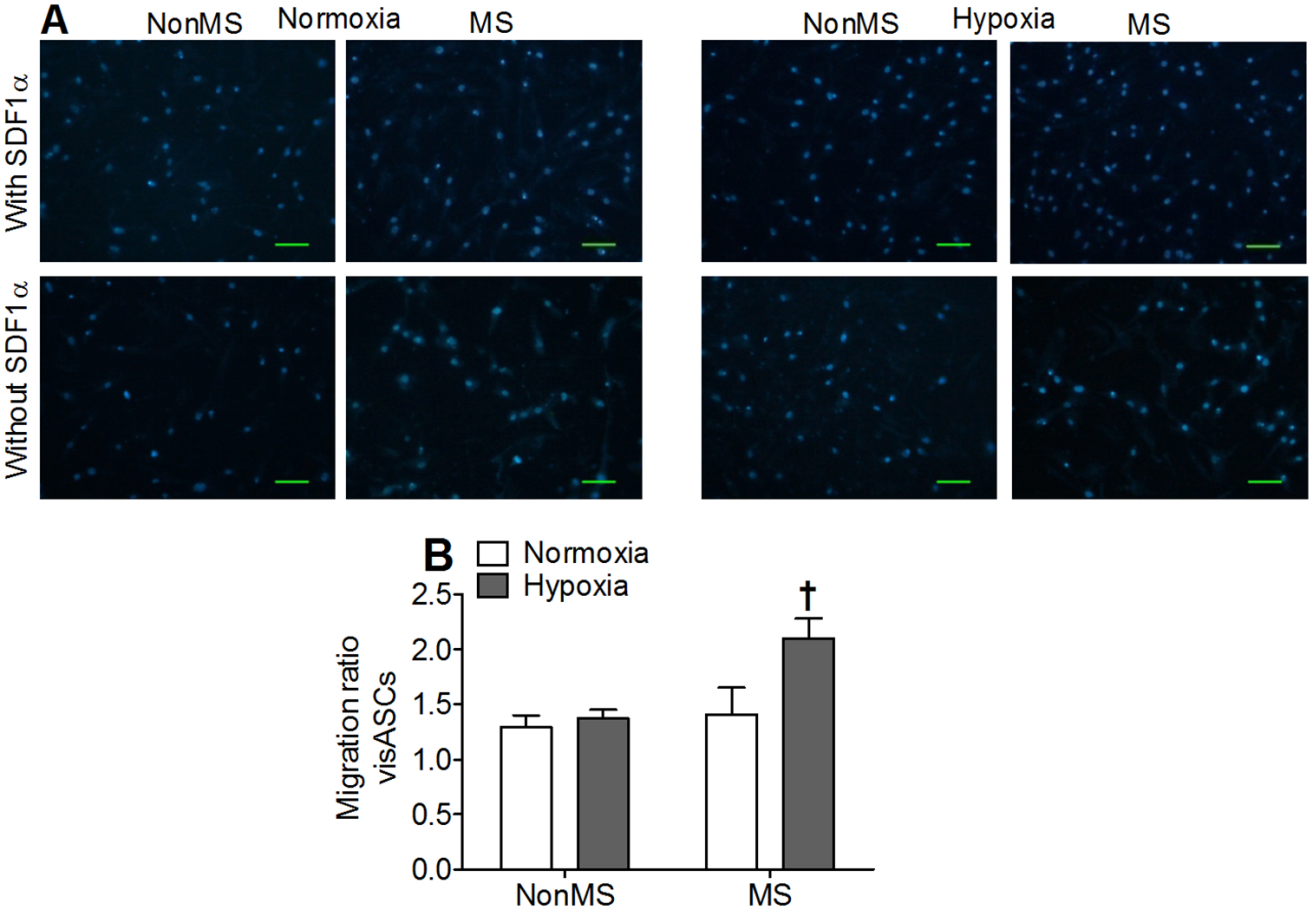
Effects of SDF1α on migration of visASCs previously cultivated under 21% O_2(g)_ or 1% O_2(g)_. **A**: Count of visASCs that migrated after 24 hours of culture toward the lower surface of the migration chamber, the compartment containing culture medium supplemented with 100 ng/mL of SDF1α or without SDF1α. Bar = 100 μm. **B**: The migration rate was calculated by dividing the number of cells detected in the presence of SDF1α by those observed in the absence of SDF1α. The results are presented as mean migration rate values ± standard error (NonMS, n = 4; MS, n = 4). **†**, significantly different results (Mann-Whitney test, P < 0.05) compared to those of NonMS subjects. NonMS: obese subjects without metabolic syndrome; MS: obese subjects with metabolic syndrome; visASC: visceral adipose tissue-derived multipotent mesenchymal cells; SDF1α: stromal cell-derived factor 1α.

VisASCs previously cultured under normoxic or hypoxic conditions were then allowed to migrate for 24 hours under the effects of the chemokine SDF1α, CD184 receptor ligand. In accordance with the percentages of CD184^+^ visASCs described by flow cytometry, the number of visASCs attracted by the SDF1α toward the bottom surface of the migration chamber was higher in MS subjects (Fig 3A). Thus, the migration rate of hypox-visASCs from MS subjects was significantly higher than the rate recorded for hypox-visASCs from NonMS subjects (Fig 3B).

### Alterations in the expression levels of NADPH oxidase family members and inflammatory cytokines in hypox-visASCs

Considering that hypoxia can increase reactive oxygen species (ROS) generation in ASCs by a nicotinamide adenine dinucleotide phosphate (NADPH) oxidase-dependent mechanism [23, 24] and that the disproportionate increase in ROS could affect the functions of ASCs [25], we evaluated the expression levels of several members of the NADPH oxidase family due to their involvement in the redox imbalance that occurs in adipose tissue during obesity [26].

**Fig 4:**
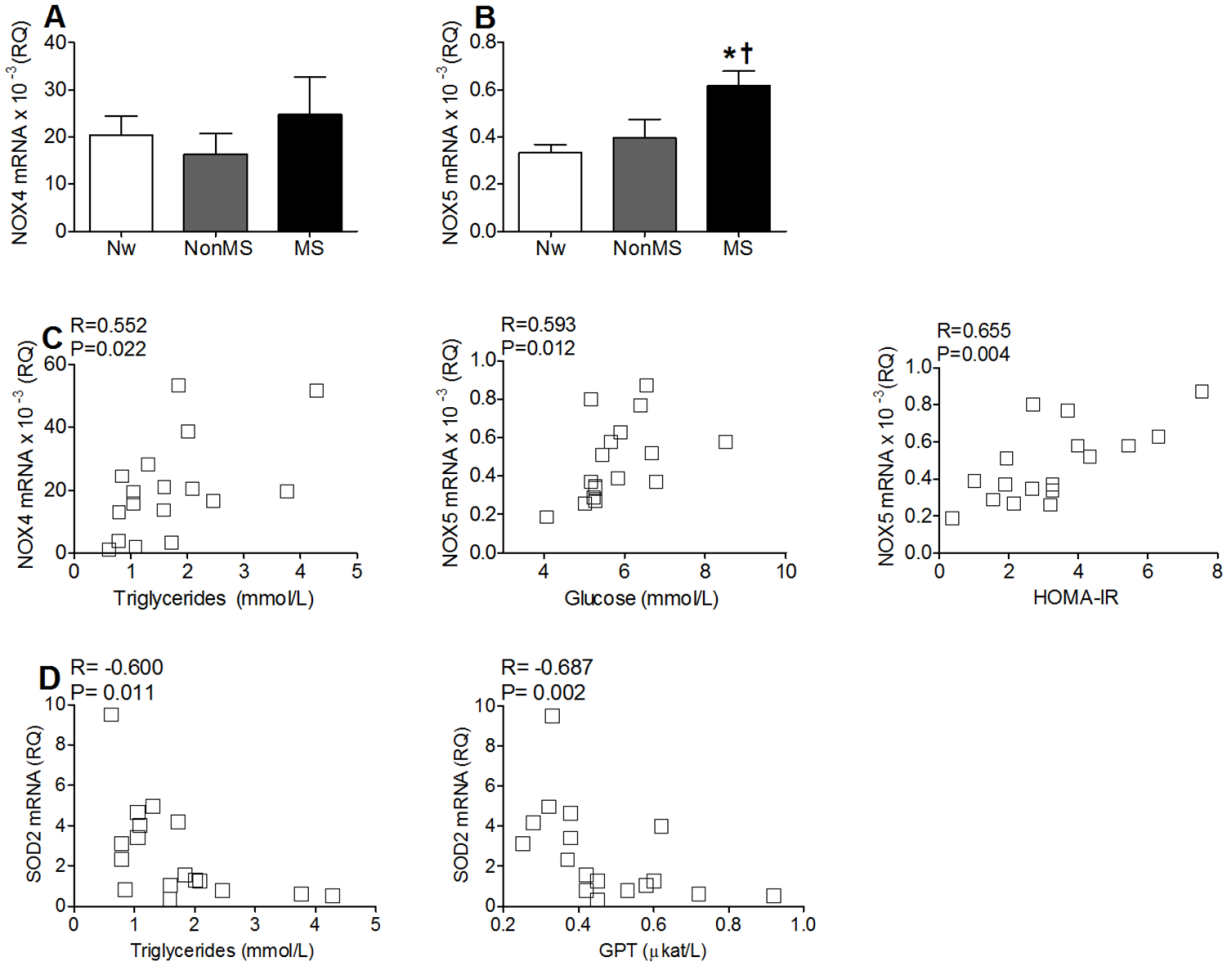
Expression of genes involved in cellular redox balance in visASCs exposed for 72 hours at 1% O_2(g)_. **A-B**: Levels of NOX4 **(A)** and NOX5 **(B)** mRNA expression in hypox-visASCs from subjects grouped by metabolic profile. **C**: Correlation between transcriptional levels of NOX4 and NOX5 mRNA with variables describing the metabolic profile of the patients. **D**: Correlation between mRNA expression levels of SOD2 in hypox-visASCs with plasma levels of triglycerides and GPT in the patients. (Nw, n = 3; NonMS, n = 7; MS, n = 7). *, Significantly different results (Mann-Whitney test; P < 0.05) to those of Nw subjects; **†**, significantly different results (Mann-Whitney test, P < 0.05) to those of NonMS subjects. Nw: normal-weight subjects; NonMS: obese subjects without metabolic syndrome; MS: obese subjects with metabolic syndrome; HOMA-IR: homeostasis model assessment to quantify insulin resistance; SOD2: superoxide dismutase 2, mitochondrial; GPT: glutamate pyruvate transaminase.

Although we observed no significant differences in NOX4 expression levels (Fig 4A), hypox-visASCs from MS subjects showed a statistically significant increase in NOX5 mRNA with respect to the other two groups of patients (Fig 4B). In addition, NOX4 and NOX5 mRNA levels increased proportionally to triglycerides, glucose concentrations and HOMA-IR of the subjects (Fig 4C). Interestingly, we also observed a negative association between transcriptional levels of SOD2 with plasma triglycerides and glutamate pyruvate transaminase (GPT) concentrations (Fig 4D).

**Fig 5:**
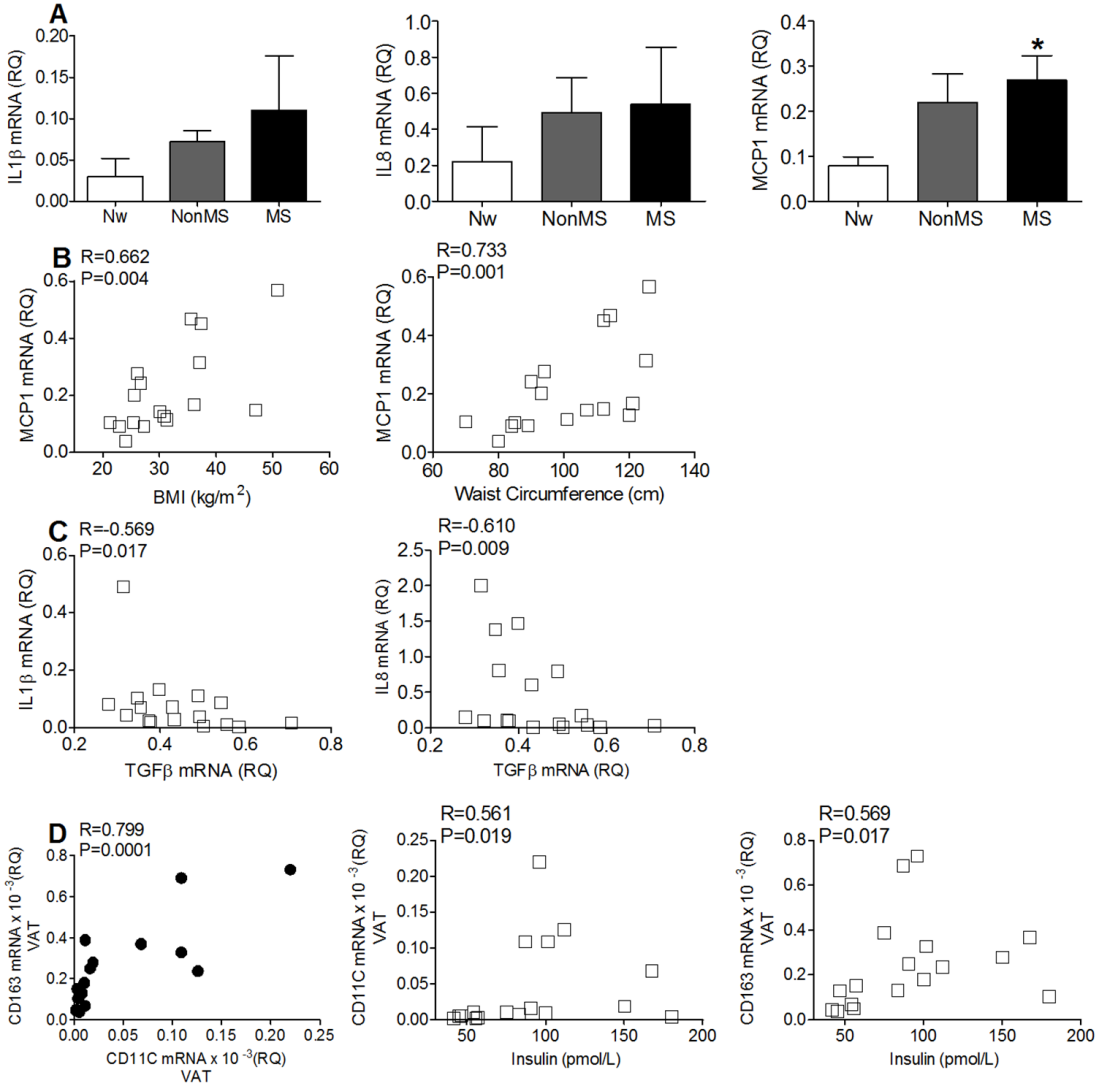
Expression of inflammatory cytokines by visASCs cultured for 72 hours at 1% O_2(g)_ and inflammatory markers in VAT. **A**: Expression levels of IL1β, IL8, and MCP1 mRNA in hypox-visASCs from subjects grouped by metabolic profile. **B**: Correlation between MCP1 expression levels with BMI and waist circumference in the patients. **C**: Correlation between levels of IL1β and IL8 mRNA with levels of TGF expressed by hypox-visASCs. **D**: Positive associations between the transcriptional levels of proinflammatory macrophage cell-surface markers CD11C and CD163 detected in VAT and plasma levels of insulin in the patients (Nw, n = 3; NonMS, n = 7; MS, n = 7). *, Significantly different results (Mann-Whitney test; P < 0.05) to those of Nw subjects. Nw: normal-weight subjects; NonMS: obese subjects without metabolic syndrome; MS: obese subjects with metabolic syndrome; BMI: body mass index; IL: interleukin; MCP1: monocyte chemoattractant protein 1; TGFβ1: transforming growth factor β1; VAT: visceral adipose tissue.

In agreement with the experiments confirming that ROS can stimulate the NF-kB pathway in adipose precursor cells [27], visASCs from MS subjects cultured for 72 hours under hypoxia showed the highest expression levels of some of the cytokines associated with the senescence-associated secretory phenotype (SASP) (Fig 5A). The levels of MCP1 mRNA, in particular, correlated positively with the BMI and waist circumference of the subjects (Fig 5B). Although no significant differences in the expression levels of IL1β and IL8 were observed (Fig 5A), the transcriptional levels of both cytokines showed a significant negative correlation with mRNA levels of the anti-inflammatory cytokine TGFβ1 (Fig 5C). Application of qPCR in the VAT enabled us to confirm a significant positive correlation between the transcriptional levels of proinflammatory macrophage cell-surface markers CD11C and CD163, and both markers with plasma insulin concentration (Fig 5D).

### Levels of VEGF and HGF secretion in visASCs under hypoxic conditions and bioactivity of hypox-visASC-conditioned medium

**Fig 6:**
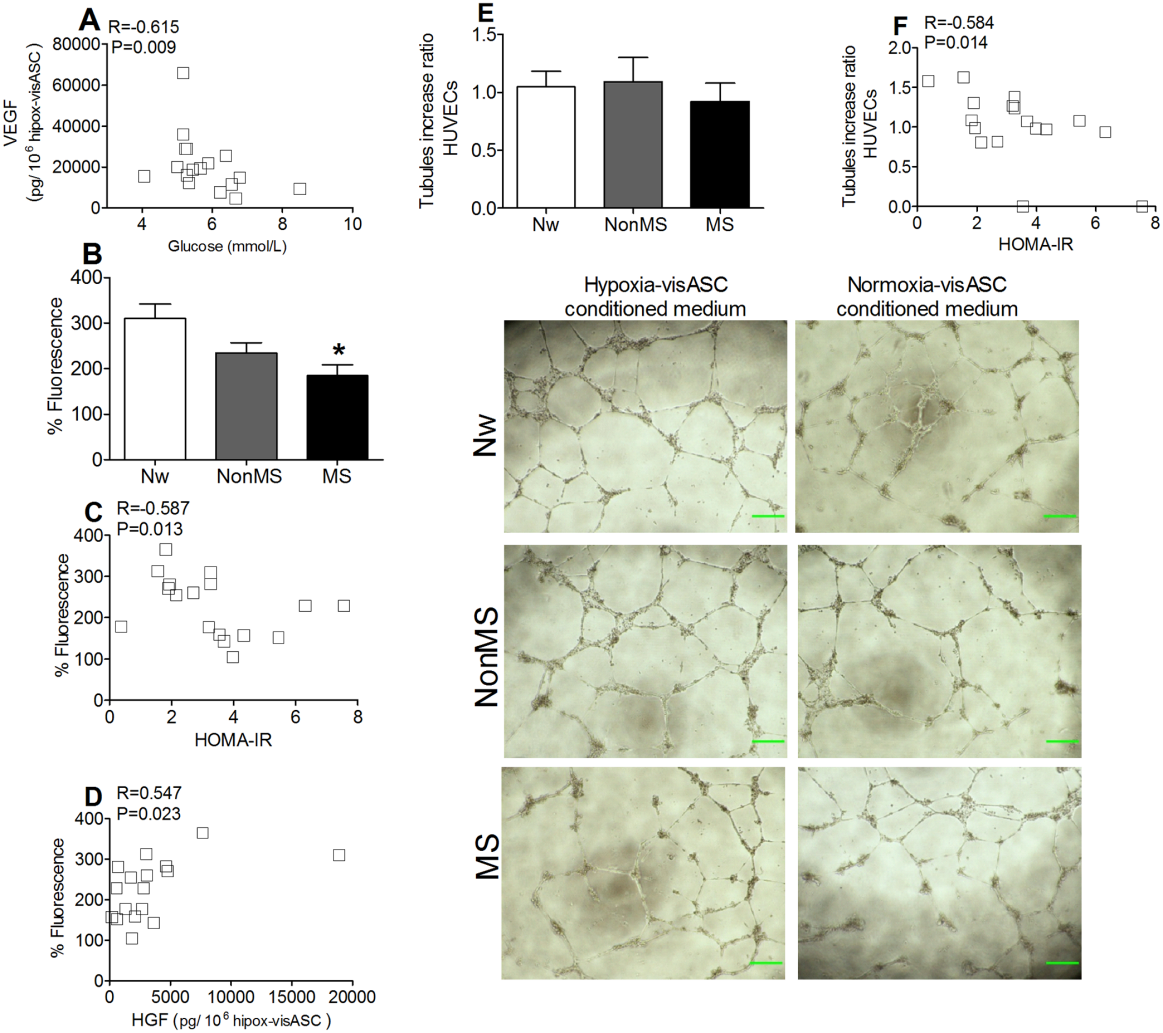
Secretion of angiogenic cytokines and bioactivity of the medium conditioned by visASCs cultured for 72 hours at 1% O_2(g)_. **A**: Correlation analysis between VEGF concentrations and plasma glucose levels in the patients. **B**: Values of fluorescence emitted by the surviving HUVECs grouped by metabolic profile. **C**: Correlation analysis between values of fluorescence emitted by the surviving HUVECs and HOMA-IR. **D**: Correlation analysis between HGF concentrations and values of fluorescence emitted by the surviving HUVECs. Concentrations of cytokines were determined by ELISA and their values normalized to 1 × 10^6^ visASCs according to the number of cells present at the time of medium collection. Cell viability was estimated by fluorescence intensity, by dividing the emitted fluorescence value in the wells where HUVECs were cultured in hypox-visASC-conditioned medium by the values emitted in the wells where HUVECs were cultured in unconditioned medium. **E**: Tubular structures and rate of increase in the length of tubules generated by HUVECs cultured in visASC-conditioned medium grouped by metabolic profile. **F**: Correlation analysis between the rate of increase in the length of tubules generated by HUVECs cultured in visASC-conditioned medium and HOMA-IR. Bar = 250 μm. The rate of increase in tubule formation was calculated by dividing the mean length of the tubules produced by HUVECs cultured in hypox-visASC-conditioned medium by the mean length of those generated by the HUVECs cultured in normo-visASC-conditioned medium. (Nw, n = 3; NonMS, n = 7; MS, n = 7). *, Significantly different results (Mann-Whitney test; P < 0.05) to those of Nw subjects. Nw: normal-weight subjects; NonMS: obese subjects without metabolic syndrome; MS: obese subjects with metabolic syndrome; HUVECs: human umbilical cord vein endothelial cells; VEGF: vascular endothelial growth factor; HGF: hepatocyte growth factor; HOMA-IR: homeostasis model assessment to quantify insulin resistance.

VEGF levels in NonMS patients tended to be greater than in the other groups (Nw = 17 515 ± 6 142 pg/10^6^cells; NonMS = 28 206 ± 6 993 pg/10^6^cells; MS = 15 353 ± 2 794 pg/10^6^cells), but did not reach statistical significance. Nor were significant differences detected in the amounts of HGF. Interestingly, the VEGF concentration in hypox-visASC-conditioned culture medium was negatively and significantly correlated with plasma glucose levels (Fig 6A).

Fig 6B shows that the survival of HUVECs cultured for 72 hours in hypox-visASC-conditioned medium from MS subjects decreased compared to that recorded in hypox-visASC-conditioned medium from Nw subjects. Indeed, HUVEC survival correlated negatively with HOMA-IR (Fig 6C) and increased proportionally to the concentration of HGF detected in hypox-visASC-conditioned medium (Fig 6D).

Although we found no significant differences in the rate of increase in tubule formation by HUVECs cultured for 24 hours in visASC-conditioned medium (Fig 6E), values showed a significant negative correlation with HOMA-IR (Fig 6F).

## Discussion

In the present study we examined, for the first time, the effects of hypoxia on the neovascular and inflammatory response of visASCs from NonMS and MS subjects. We observed an increased capacity for tubule formation by hypox-visASCs from NonMS subjects compared to Nw and MS subjects, which was accompanied by statistically significant differences in the percentage of CD140b^+^ cells, the migration rate and the levels of NOX5 and MCP1 mRNA expression.

Previous experiments have confirmed that the exposure of human ASCs to 10 minutes of hypoxia increases ROS generation by means of NADPH oxidase activity and promotes CD140b receptor activation [23, 24]. More recently it has been reported that platelet-derived growth factor-D (PDGF-D), CD140b receptor ligand, enhances generation of mitochondrial ROS [28]. According to these studies, activation of the CD140b receptor could influence cell redox balance in human ASCs, since it is a target of ROS generated by hypoxia-induced NADPH oxidase activity. Moreover, its PDGF-D ligand-dependent activation favors the production of mitochondrial ROS. Considering the harmful effects of redox imbalance for precursor cells [29, 30], it seems possible that the significant decrease in the percentage of CD140b^+^ cells and NOX5 mRNA observed in hypox-visASCs from NonMS subjects contributed to the differences in tubule formation capacity between the visASCs of the two groups of obese subjects.

In line with a possible NF-kB signaling activation by the redox imbalance which underlies metabolic disorders [27; 31], our laboratory has previously revealed increased levels of NFκB in visceral adipose tissue [32] as well as an increased percentage of senescent visASCs from morbidly obese patients [33] and the present study also corroborated alterations in the expression levels of cytokines linked to the SASP. In fact, expression levels of MCP1 increased significantly with increasing BMI and waist circumference in the patients. As experiments in genetically engineered mice have shown that increased levels of MCP1 promote macrophage infiltration into adipose tissue and the appearance of the insulin-resistant phenotype [34, 35], it is likely that visASCs contribute to the worsening inflammatory state seen in the visceral adipose tissue of obese insulin-resistant subjects compared to insulin-sensitive subjects [36–39]. Concordantly, hypox-visASCs from MS subjects showed significantly higher MCP1 mRNA levels than those expressed by Nw subjects and previous experiments have suggested that changes in the production of MCP1 in visceral adipose tissue could precede increased immune cell infiltration in patients with early metabolic dysfunction [40]. It is interesting to note that the increased expression levels of IL8 and IL1β correlated inversely with those of TGFβ1, a cytokine involved in controlling the immune response [41].

Consistent with the possibility that visASCs actively contribute to increased tissue inflammation, *in vitro* experiments have revealed that they are more proinflammatory than those from subcutaneous adipose tissue [42]. In addition, human preadipocytes exposed to hypoxia secrete higher levels of inflammatory cytokines, and their conditioned medium causes a greater increase in the expression of intercellular adhesion molecule-1 and greater adhesion of monocytes to endothelial cells than adipocytes [43]. The higher percentage of CD140b^+^CD44^+^ hypox-visASCs observed in MS subjects suggests the possible involvement of ASCs in the recruitment of monocytes, as it has been reported that CD44 is involved in the sequestering of monocytes into inflamed tissues [44]. Hypox-visASCs from MS subjects also showed a greater capacity for migration from the chemotactic effects of SDF1α, confirming that these cells could be mobilized by the SDF1α secreted by endothelial cells [45].

While the chemotactic effects of SDF1α have been linked to increased angiogenic potential in human ASCs subjected to hypoxia [6], our results indicate otherwise for the particular case of hypox-visASCs from obese subjects, as the increased triglyceride and plasma cholesterol levels were associated with an increased percentage of CD140b^+^CD184^+^ hypox-visASCs. Although MS subjects had a higher percentage of CD140b^+^CD184^+^ hypox-visASCs than NonMS subjects, the survival of HUVECs cultured in medium conditioned by hypox-visASCs from MS subjects significantly decreased compared with those grown in medium conditioned by hypox-visASCs from Nw subjects. Furthermore, the concentration of VEGF secreted by hypox-visASCs and the tubule formation induced by its conditioned medium negatively correlated with plasma glucose levels and insulin resistance, respectively

The significant positive correlation between the survival of HUVECs and HGF concentration detected in hypox-visASC-conditioned medium suggests that HGF likely influenced cell survival. Indeed, the application of RNA interference techniques has shown that deletion of HGF in ASCs leads to decreased survival and proliferation of endothelial lineage cells cultured in their conditioned medium [46].

**Fig 7:**
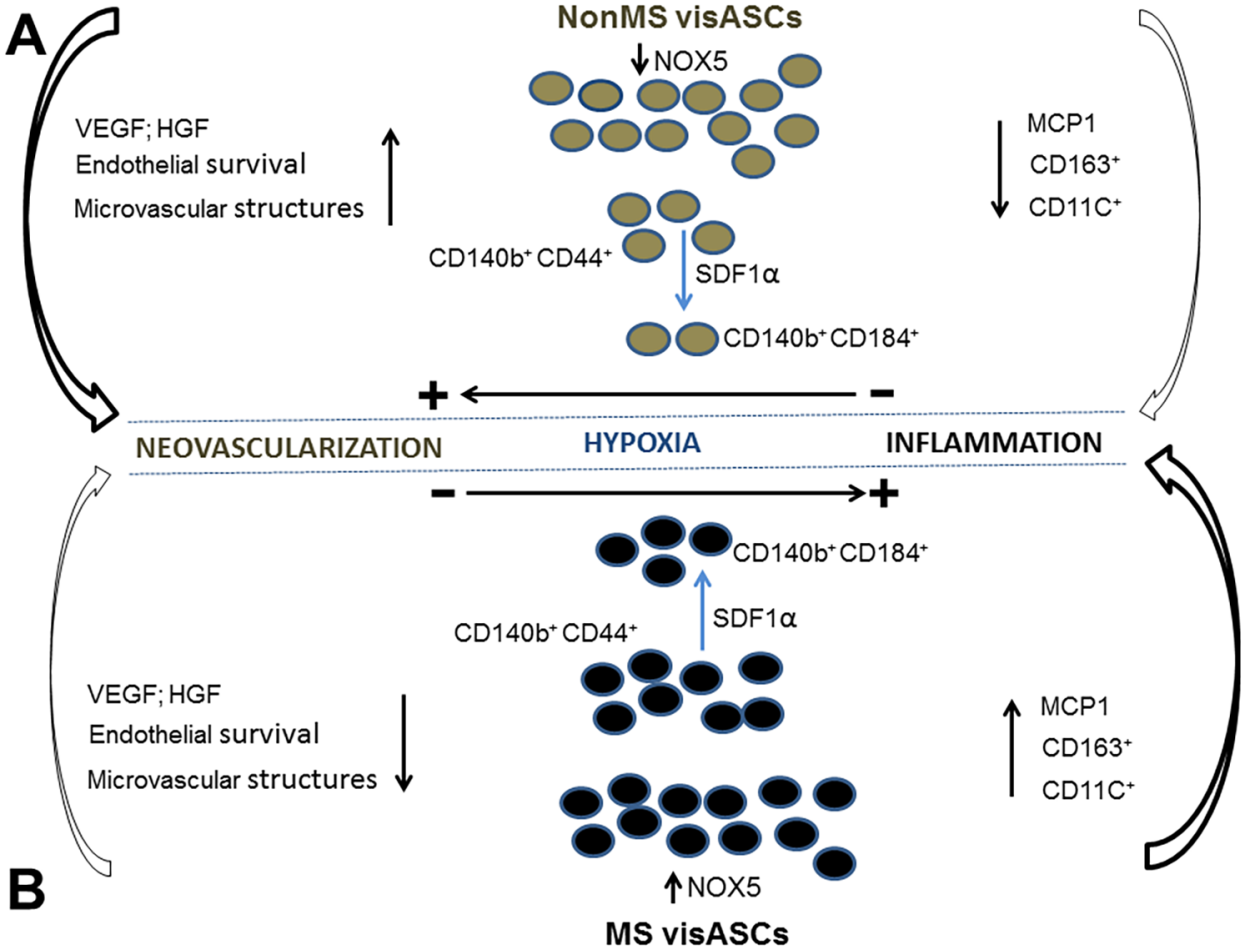
Different response of visASCs to a micro-local oxygen concentration decrease in metabolically active sites of the adipose tissue. **A**: Hypox-visASCs from NonMS subjects, characterized by a higher redox balance associated with lower NOX5 expression levels, migrate in response to the chemotactic effects of SDF1α liberated during hypoxia. Hypox-visASCs from NonMS subjects directly contribute to or stimulate the formation of microvascular structures through the secretion of angiogenic cytokines, such as VEGF, while maintaining proinflammatory cytokine secretion, such as MCP1, under control. **B**: Hypox-visASCs from MS patients, characterized by a lower redox balance associated with higher NOX5 expression levels, could have a more active migratory behavior in relation to SDF1α. However, hypox-visASCs from MS patients show a lower ability to actively participate in or stimulate the formation of microvascular structures, while they favor the accumulation of CD11C^+^ and CD163^+^ macrophages in the adipose tissue through their contribution to the increased tissue levels of MCP1. ↑ Increased response; **↓** Decreased response; → Hypox-visASCs main response NonMS: obese subjects without metabolic syndrome; MS: obese subjects with metabolic syndrome; visASC: visceral adipose tissue-derived multipotent mesenchymal cells; hypox-visASCs: visASC under hypoxia; NOX5: NADPH Oxidase 5; SDF1α: stromal cell-derived factor 1α; VEGF: vascular endothelial growth factor; MCP1: monocyte chemoattractant protein 1; CD11C^+^, CD163^+^: proinflammatory macrophage cell-surface markers.

Taken overall, our study indicates that visASCs from obese subjects with and without metabolic syndrome show a different behavioral response to hypoxia that could have consequences for adipose tissue functionality (Fig 7). Thus, it is likely that the preservation of neovascular properties in the visASCs from the NonMS subjects encourages healthy adipose tissue expansion in response to excess caloric intake. However, the hypox-visASCs from the MS subjects show an inflammatory predisposition, to the detriment of their neovascular function, which could contribute to the decreased angiogenic potential and increased inflammation underlying adipose tissue dysfunction in obesity. This study also indicates that visASCs could be implicated in the pathogenesis of metabolic syndrome since we confirmed that the deterioration in the metabolic profile of the subjects was accompanied by decreased expression levels of SOD2 as well as increased expression levels of NADPH oxidase enzymes and inflammatory cytokines in hypox-visASCs. In addition, the study findings suggest that the therapeutic use of ASCs from MS subjects could be limited due to the significant decrease in their neovascular function and support the importance of taking into account not only the BMI but also the metabolic profile of the subjects during the implementation of ASC-based therapy to promote neovascularization.

## Supporting information

S1 Fig Study design in order to examine the neovascular properties of hypox-visASCs from normal-weight subjects and obese patients with and without metabolic syndrome. Every biopsy sample was divided into one piece immediately frozen in liquid nitrogen and stored at -80°C until posterior analysis by qPCR; and another piece was immediately processed by enzymatic digestion to isolation and expansion of stromal vascular fraction derived from greater omentum adipose tissue. VisASCs cultured under normoxic or hypoxic conditions were characterized by tubule formation assay, flow cytometry, migration rate, and qRT-PCR. ELISA kit was used to quantify VEGF and HGF, and the effects of visASCs-conditioned medium on survival and endothelial cell tubule formation were evaluated. Hypox-visASCs: visceral adipose tissue-derived multipotent mesenchymal cells cultured under hypoxic conditions; SDF1α: stromal cell-derived factor 1α; IL: interleukin; MCP1: monocyte chemoattractant protein 1; TGFβ1: transforming growth factor β1; HUVECs: human umbilical cord vein endothelial cells; VEGF: vascular endothelial growth factor; HGF: hepatocyte growth factor.
(TIF)
