# Supplementary figures and images for: Different response to hypoxia of adipose-derived multipotent cells from obese subjects with and without metabolic syndrome
Source: PLoS One. 2017 Nov 22;12(11):e0188324. doi: 10.1371/journal.pone.0188324 (PMC5699836; doi:10.1371/journal.pone.0188324)

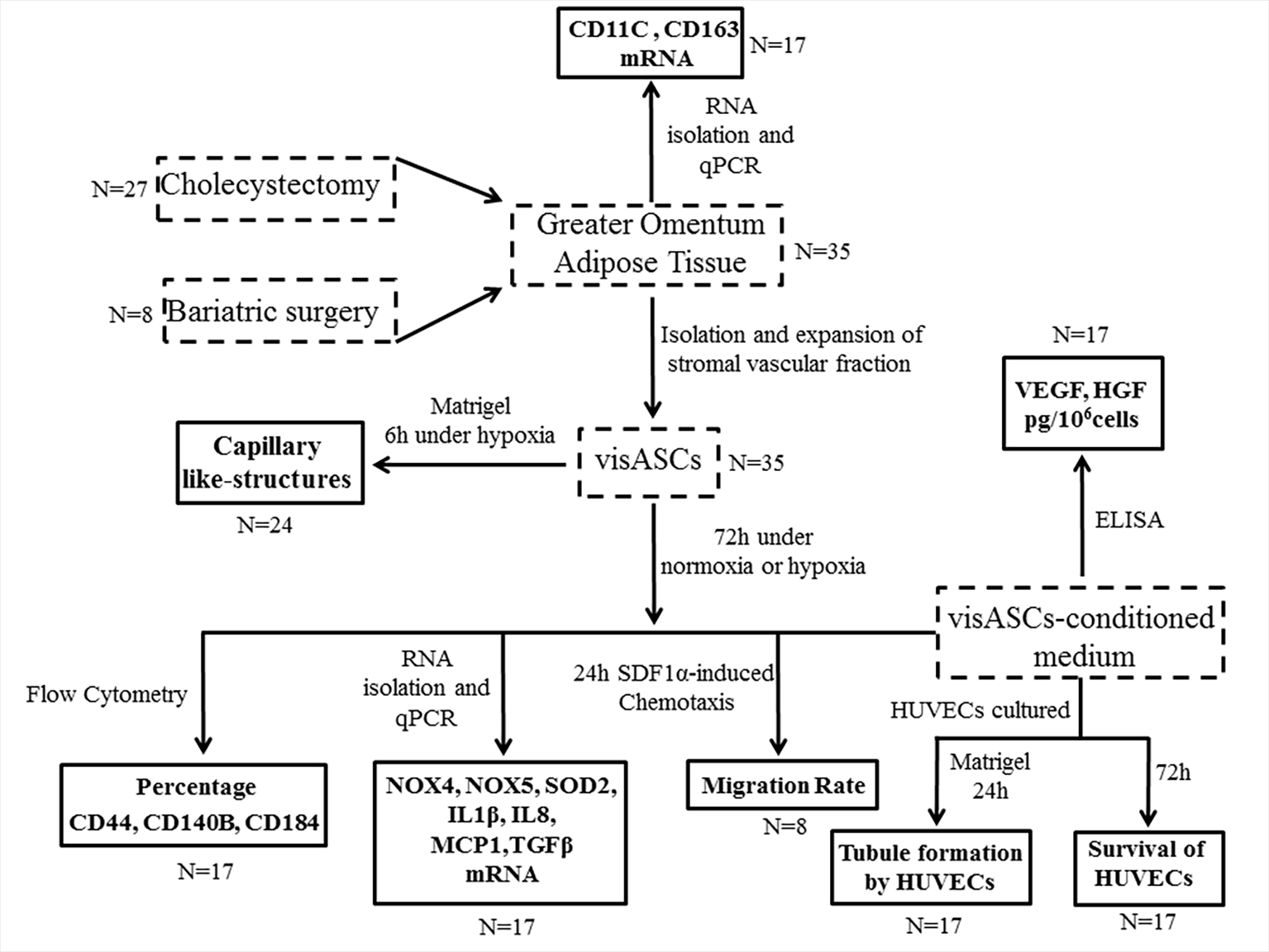

Supplement: S1 Fig — Every biopsy sample was divided into one piece immediately frozen in liquid nitrogen and stored at -80°C until posterior analysis by qPCR; and another piece was immediately processed by enzymatic digestion to isolation and expansion of stromal vascular fraction derived from greater omentum adipose tissue. VisASCs cultured under normoxic or hypoxic conditions were characterized by tubule formation assay, flow cytometry, migration rate, and qRT-PCR. ELISA kit was used to quantify VEGF and HGF, and the effects of visASCs-conditioned medium on survival and endothelial cell tubule formation were evaluated. Hypox-visASCs: visceral adipose tissue-derived multipotent mesenchymal cells cultured under hypoxic conditions; SDF1α: stromal cell-derived factor 1α; IL: interleukin; MCP1: monocyte chemoattractant protein 1; TGFβ1: transforming growth factor β1; HUVECs: human umbilical cord vein endothelial cells; VEGF: vascular endothelial growth factor; HGF: hepatocyte growth factor. (TIF) [file pone.0188324.s001.tif]
